# Global trends and research hotspots in the treatment of mental disorders with transcranial magnetic stimulation: a bibliometric analysis

**DOI:** 10.3389/fpsyt.2025.1526225

**Published:** 2025-06-04

**Authors:** Zong-ke Ma, Yi-xin Wei, Ling Rao, Bao-jin Li, Qiang Gao

**Affiliations:** ^1^ Rehabilitation Medicine Center and Institute of Rehabilitation Medicine, West China Hospital, Sichuan University, Chengdu, China; ^2^ Key Laboratory of Rehabilitation Medicine in Sichuan Province, West China Hospital, Sichuan University, Chengdu, China

**Keywords:** transcranial Magnetic Stimulation, mental disorders, depression, obsessive-compulsive disorder, safety, bibliometric analysis, trends and hotspots

## Abstract

**Background:**

Transcranial Magnetic Stimulation (TMS) is increasingly utilized in the treatment of mental disorders (MD). The exploration and expanding application of various new TMS mode have significantly propelled the advancement of related clinical research.

**Methods:**

We reviewed research published in the Science Citation Index Expanded of Web of Science Core Collection database. Using Citespace 6.1, Vosviewer 1.6.20, and Scimago Graphica 1.0.38 software, we conducted a comprehensive visual analysis of TMS on MD from multiple dimensions, including influential countries/regions, institutions, authors, and high-frequency keywords and burst keywords.

**Results:**

A total of 611 papers between 1996 and 2023 were identified. Recently, the application of TMS on MD have gained increasing recognition. The USA leads in research publications in this field, followed by Germany and China. Institutionally, the University of Toronto in Canada ranks first (n=48); Professor Zafiris J. Daskalakis from the University of California tops among individual researchers (n=24). Cluster analysis of keywords reveal four representative clusters, demonstrating shifts in research focus and direction over time. Current hotspots focus on exploring the effectiveness of different TMS modes and stimulation targets in treating severe depression, obsessive-compulsive disorder, and schizophrenia. Analysis of burst keywords indicated that the latest research are the feasibility and safety of various emerging TMS stimulation mode for treating refractory depression, obsessive-compulsive disorder, negative symptoms of schizophrenia.

**Conclusions:**

Our study provides valuable insights into the current hotspots and emerging trends of TMS in the treatment of MD, providing a direction for future research to consider.

## 1 Introduction

Mental disorder (MD) are syndromes characterized by clinically significant disturbance in an individual’s cognition, emotional regulation, or behavior that reflects a dysfunction in the psychological, biological, or developmental processes that underlie mental and behavioral functioning (1). According to the WHO’s 2019 report, 970 million people worldwide suffered from MD, meaning 1 in 8 individuals was affected (2). Among these, depression and anxiety are the most common, followed by obsessive-compulsive disorder, post-traumatic stress disorder, and substance addiction. Together, these conditions account for 7.4% of the global disease burden, contributing to lowered work productivity, family dysfunction, substance misuse, suicide, and reduced life expectancy (3).

The current treatment methods for MD primarily include pharmacotherapy, psychotherapy, and neuromodulation (4). Pharmacotherapy for MD employs a variety of mechanisms depending on the specific condition being treated (5). However, the overall effectiveness of most antipsychotic drugs on the market is limited, and their safety and tolerability remain concerns, with common side effects including sedation, cognitive decline, dystonia, tardive dyskinesia, and metabolic syndrome (6). The most commonly used psychotherapy is cognitive-behavioral therapy (CBT), a directive approach that uses behavioral and linguistic techniques to identify and correct negative thoughts (7). CBT has varying efficacy in treating anxiety (8), depression (9), obsessive-compulsive disorder (OCD) (10),and PTSD (post-traumatic stress disorder) (11). However, some overlooked negative effects exist in psychological treatment (12), such as behavioral therapy reinforcing poor interpersonal communication patterns and long-term psychotherapy reducing the independent judgment of patients with MD (13).

Given these challenges, non-invasive neuromodulation is regarded as a promising treatment option, with Transcranial Magnetic Stimulation (TMS) emerging as a widely adopted method due to its favorable safety profile and patient tolerability (14). Since its introduction by Antony Barker in 1985 (15), its application has expanded rapidly, transitioning from basic neuroscience research to clinical interventions for neuropsychiatric disorders over the past three decades (16, 17). TMS operates by delivering repetitive electromagnetic pulses through a scalp-placed coil, generating magnetic fields (approximately 1.5 Tesla) that depolarize neurons beneath the cortex, thereby altering cortical excitability and connectivity (18, 19). Protocols such as repetitive TMS (rTMS) and theta burst stimulation (TBS) have shown efficacy in treating MD (18), yet the field remains challenged by inconsistent outcomes, variable protocol effectiveness, and a lack of consensus on optimal stimulation parameters—such as frequency, intensity, and target selection (19). These gaps highlight the urgent need for a comprehensive synthesis of the global research landscape to distill actionable insights and guide future advancements.

This study addresses this gap through a bibliometric analysis of TMS applications for MD, covering publications from 1975 to 2023 in the Web of Science Core Collection (WoSCC) database. By employing visualization tools such as CiteSpace, VOSviewer, and Scimago Graphica (20), we aim to map the global research landscape, identify influential contributors, and highlight current hotspots and emerging trends. This analysis is crucial for optimizing TMS treatment by providing clinicians with a clearer understanding of effective stimulation parameters, target regions, and safety considerations, while offering researchers a foundation to refine protocols and explore new applications. For instance, understanding shifts in research focus—such as the increasing emphasis on TBS or deep TMS (dTMS)—can inform the development of more precise, evidence-based interventions, ultimately improving patient outcomes and fostering international collaboration to address disparities in research capacity between developed and developing regions.

## 2 Materials and methods

### 2.1 Data sources and search strategy

Publications with related themes from the 1975 to 2023 were searched from the Science Citation Index Expanded (SCIE) of the WoSCC database on 26th February 2024. To obtain documents explicitly employing the concerning terms we performed a topical search with the query TS= (“Transcranial Magnetic Stimulation” OR magnetic field therap* OR “Magnetic Stimulation, Transcranial” OR “Magnetic Stimulations, Transcranial” OR “Stimulation, Transcranial Magnetic” OR “Stimulations, Transcranial Magnetic” OR “Transcranial Magnetic Stimulations” OR “Transcranial Magnetic Stimulation, Single Pulse “ OR “Transcranial Magnetic Stimulation, Paired Pulse” OR “Transcranial Magnetic Stimulation, Repetitive” OR “noninvasive brain stimulation” OR TMS) AND TS= (“Mental Disorder” OR “Psychiatric Illness” OR “Psychiatric Illnesses” OR “Psychiatric Diseases” OR “Psychiatric Disease” OR “Mental Illness” OR “Illness, Mental” OR “Mental Illnesses” OR “Psychiatric Disorders” OR “Psychiatric Disorder” OR “Behavior Disorders” OR “Diagnosis, Psychiatric” OR “Psychiatric Diagnosis” OR “MD, Severe” OR “Mental Disorder, Severe” OR “Severe Mental Disorder” OR “Severe MD”). We only selected articles or reviews in English, other document types, such as proceeding paper, bookchapter, and retracted paper, were excluded. Finally, a total of 882 literature records were included.

### 2.2 Data extraction and analysis

**Figure 1:**
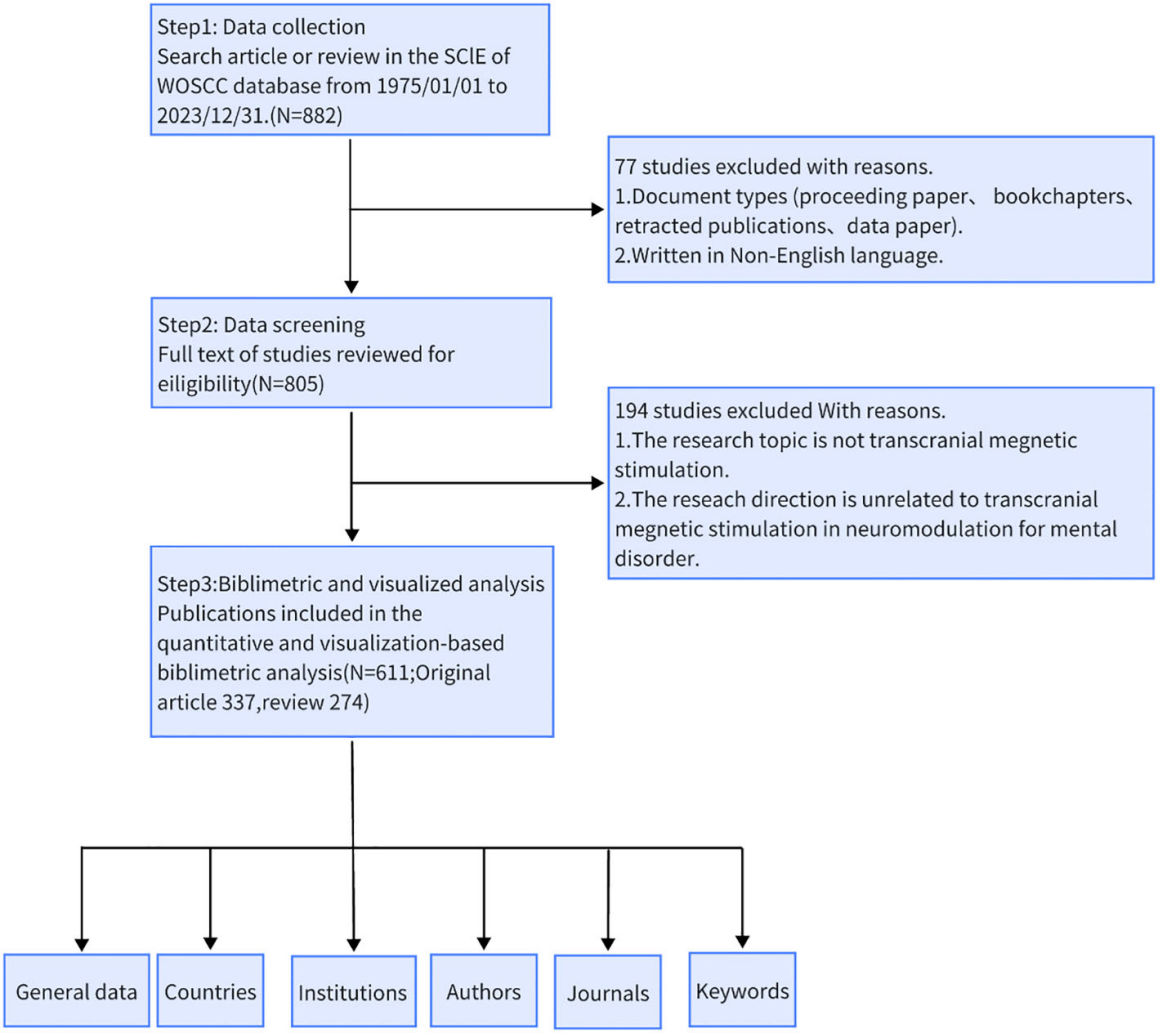
Flow chart of search strategy and analysis process.

All included documents underwent peer review. Bibliometric data were imported into Endnote 20 for deduplication, after two authors (ZK-M and LR) independently screened the titles, abstracts, and full texts of the included papers to identify eligible studies based on the exclusion criteria. The exclusion criteria were as follows (1): The research topic did not involve transcranial magnetic stimulation (2); The study was unrelated to the use of transcranial magnetic stimulation in neuromodulation for MD. Ultimately, we included 611 articles (337 original articles, 274 reviews) that met the inclusion criteria. The procedures for data collection and retrieval are illustrated in **Figure 1**.

## 3 Result

### 3.1 Annual global publication outputs and growth trend

**Figure 2:**
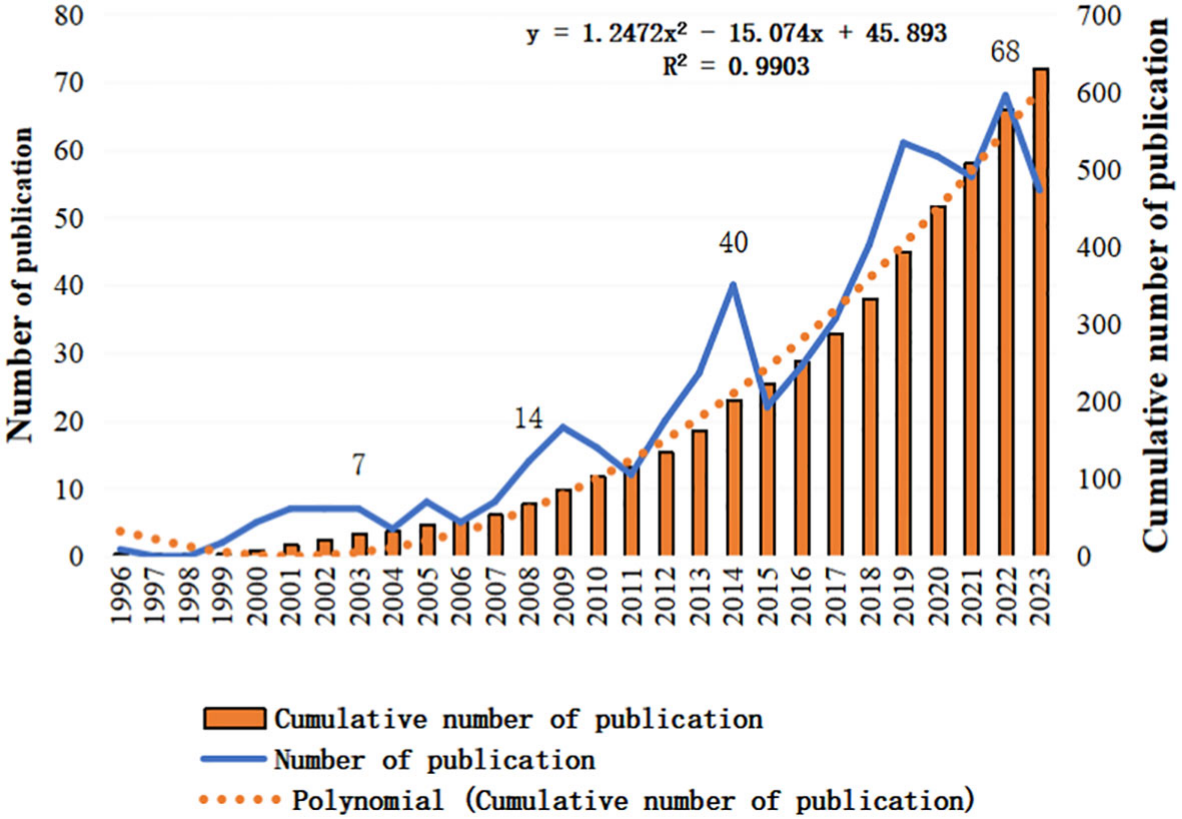
Trend of publication output from 1996 to 2023 on TMS for MD.

The initial search of the WoSCC database identified 882 articles. After excluding non-English papers, unrelated topics, and other document types, 611 articles were selected for final analysis, including 337 original research articles and 274 reviews. The first article on TMS treatment for MD was published in 1996 by Conca A (21), reporting that TMS intervention could alleviate symptoms in patients with depression. The annual global publication output and growth trend from 1996 to 2023 are illustrated in **Figure 2**. The publication timeline can be divided into three distinct phases: the initial stage (1996-2007), the slow growth stage (2008-2016), and the rapid growth stage (2017-2023). In the initial stage, annual publications were consistently below 10. During the slow-growth stage, apart from 2014, annual publications was below 30 over these 9 years. In the rapid growth stage, particularly from 2019 to 2023, a significant rise in anxiety and depression cases due to the COVID-19 pandemic (22) contributed to annual publications consistently exceeding 30, peaking at 68 publications in 2022. Additionally, linear regression analysis demonstrated a strong positive correlation between the cumulative annual publication count and the publication year ( $Y=1.1798x^{2}-13.646x+40.887,\;R^{2}=0.9925$ suggesting that research in this field is likely to continue expanding in the future.

### 3.2 Countries/regions distribution

**Figure 3:**
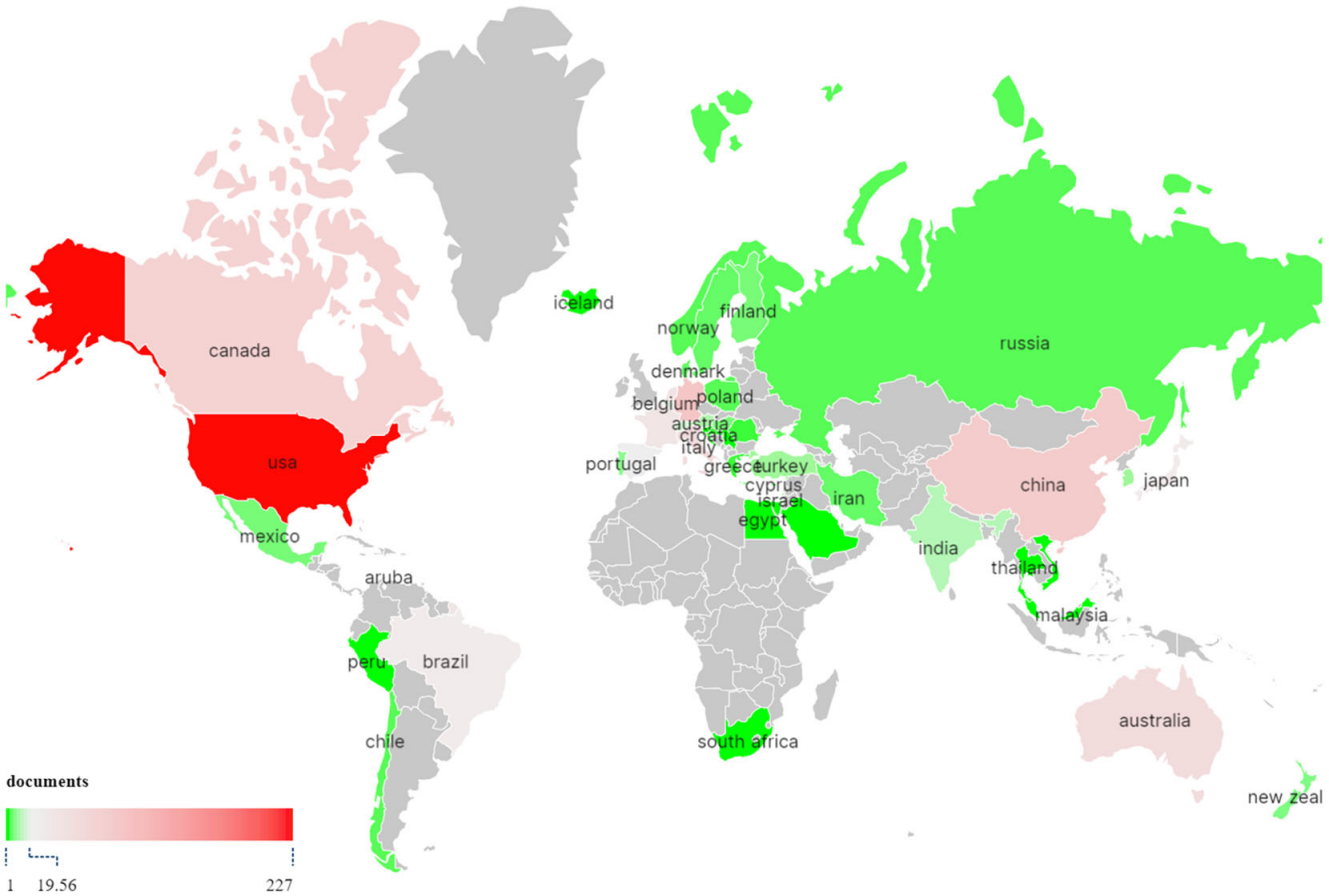
Distribution of countries/regions on TMS for MD.

**Figure 4:**
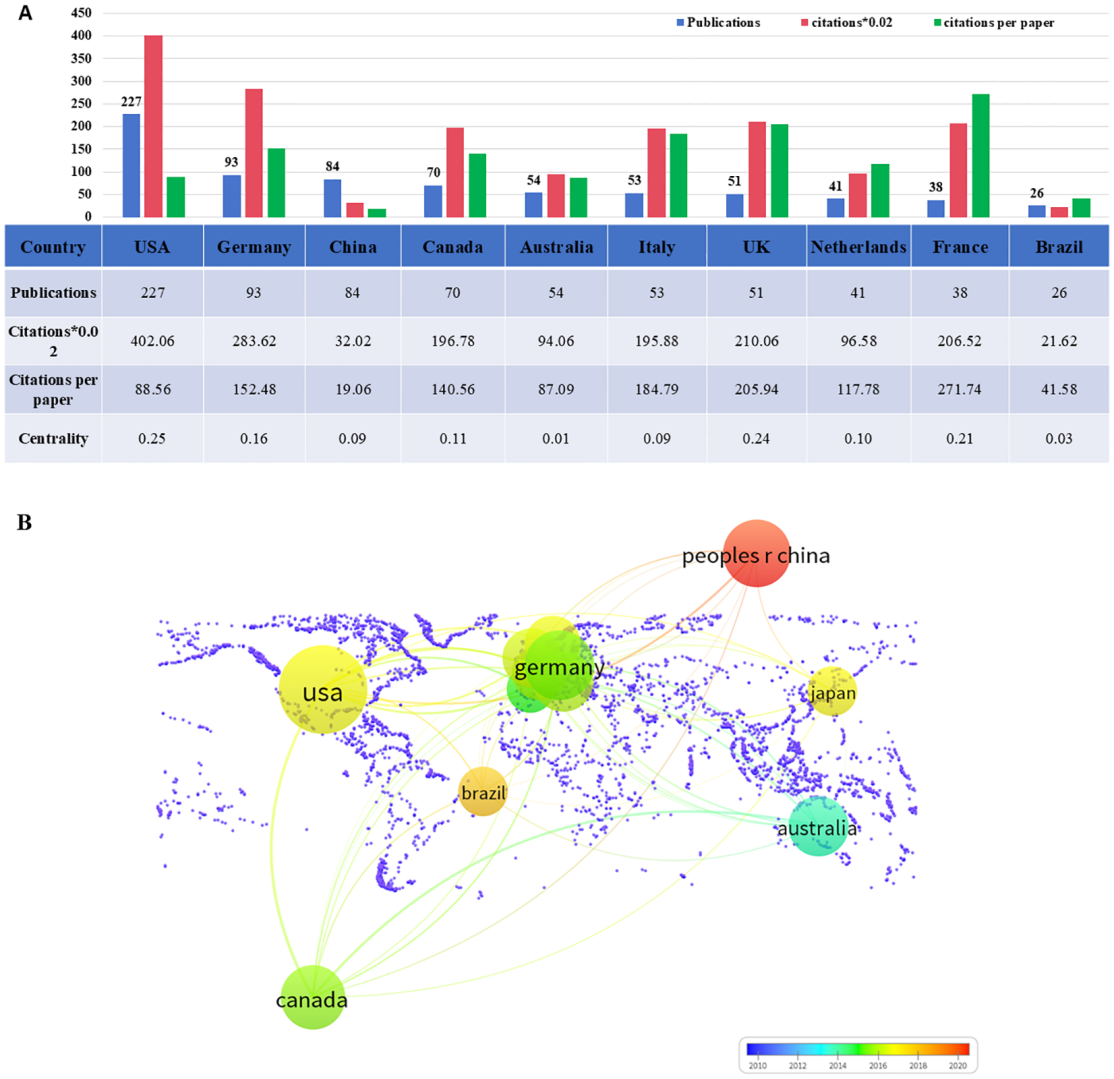
The top 10 countries and their cooperative network on TMS for MD. **(A)** The number of publications, total citations, citations per paper and centrality of the top 10 countries; **(B)** The co-operative network visualization map of top 10 countries/regions on TMS for MD.

**Figure 5:**
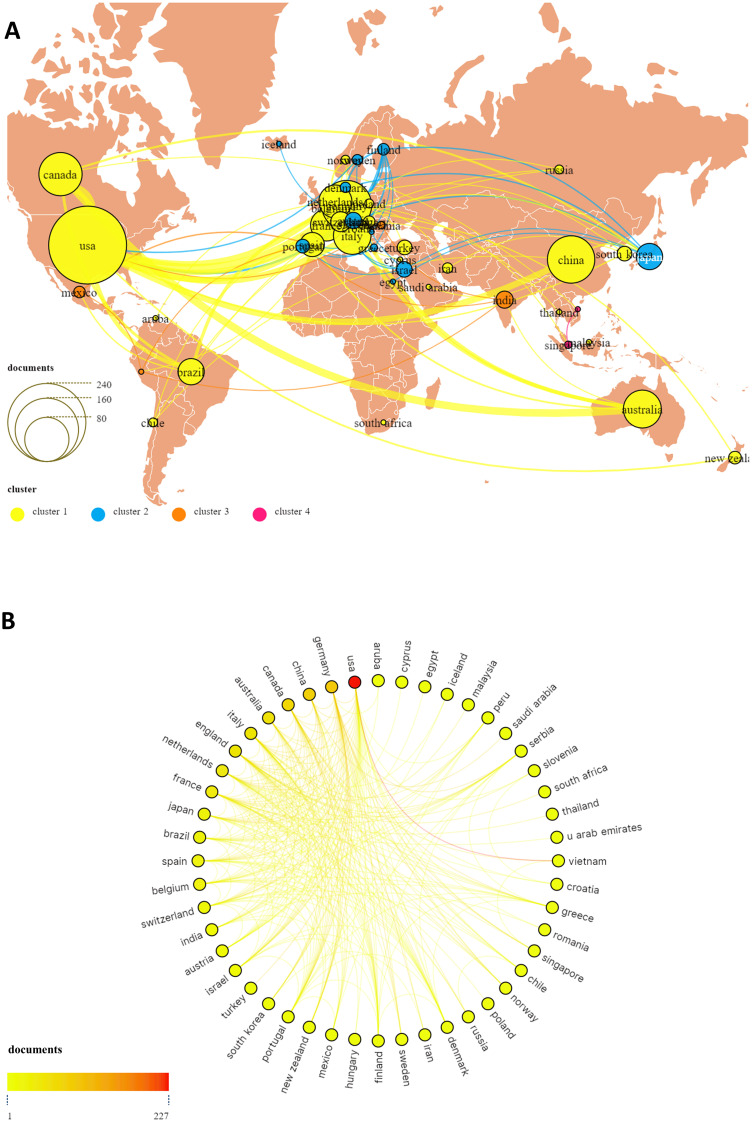
The publications and co-operative network visualization map of all countries/regions on TMS for MD **(A, B)**.

TMS interventions for MD have been conducted across 53 countries or regions worldwide, as illustrated in **Figure 3**. **Figure 4** highlights the top 10 countries based on publication output (**Figure 4A**) and international collaboration (**Figure 4B**). The USA leads with the highest number of publications (227, 37.2%), followed by Germany (93, 15.2%) and China (84, 13.7%). In terms of citation impact, the USA also ranks first with 20,101 total citations, followed by Germany (14,181) and the United Kingdom (10,503). A cooperation analysis of countries and regions, performed using VOSviewer and Scimago Graphica, elucidates the global collaboration networks and their publication contributions in this field. As depicted in **Figure 5**, the collaboration network encompasses 48 of the 53 countries/regions and is organized into four distinct clusters, each denoted by a unique color. The largest cluster, shown in yellow, comprises 22 countries and is centered around the UK, Germany, and China. The USA exhibits the most extensive collaboration network, with 33 partner countries, followed by the UK, France, and Germany, each with 28 partners. **Figure 5A** further illustrates the publication volume of the 53 countries, with larger circle sizes indicating higher output, while **Figure 5B** employs a color gradient—from yellow to red—where redder hues signify greater publication volume.

### 3.3 Institutions analysis

**Figure 6:**
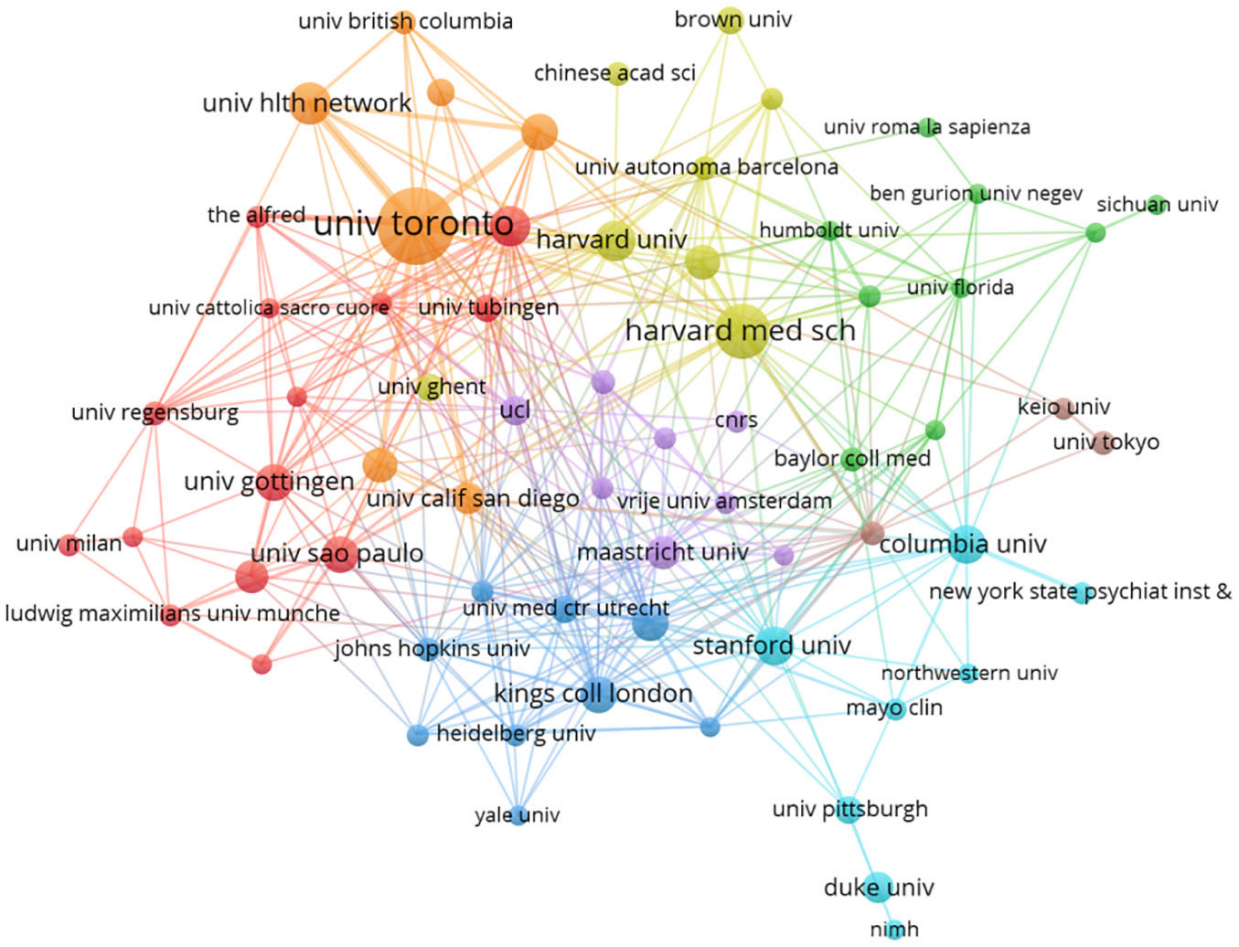
The cooperative network map of institutions on TMS for MD.

A total of 1,172 research institutions worldwide are involved in TMS treatment research for MD. The **Table 1** highlights the top 10 institutions based on their publications, with a significant concentration in the USA and Canada. The majority of the publications originate from the University of Toronto and University Health Network in Canada, along with Harvard Medical School and Harvard University in the United States. Collectively, these top ten institutions have published 196 papers, accounting for 32.08% of the total output. Notably, Harvard University has the highest average citations per paper (n=247.38). A collaboration network among these institutions was visualized using VOSviewer, focusing on those with at least 5 published papers. The resulting map, as depicted in the **Figure 6**, includes 67 research institutions connected by 364 links, organized into 8 clusters. The largest cluster, marked in red, comprises 14 institutions, with centering on Monash University, University São Paulo, and the University of Göttingen.

**Table 1:** The top 10 productive institutions regarding the research on TMS for MD.

Rank	Organization	Country	Publications	Citations	Citations per paper
1	University of Toronto	Canada	48	4742	98.79
2	Harvard Medical School	America	27	852	31.56
3	University Health Network	Canada	17	1326	78.00
4	Harvard University	America	16	3958	247.38
5	Monash University	Australia	16	2974	185.88
6	Columbia University	America	15	1342	89.47
7	Stanford University	America	15	439	29.27
8	ctr addict & mental health	Canada	14	814	58.14
9	Kings college London	England	14	2205	157.50
10	University São Paulo	Brazil	14	594	42.43

### 3.4 Author analysis

**Figure 7:**
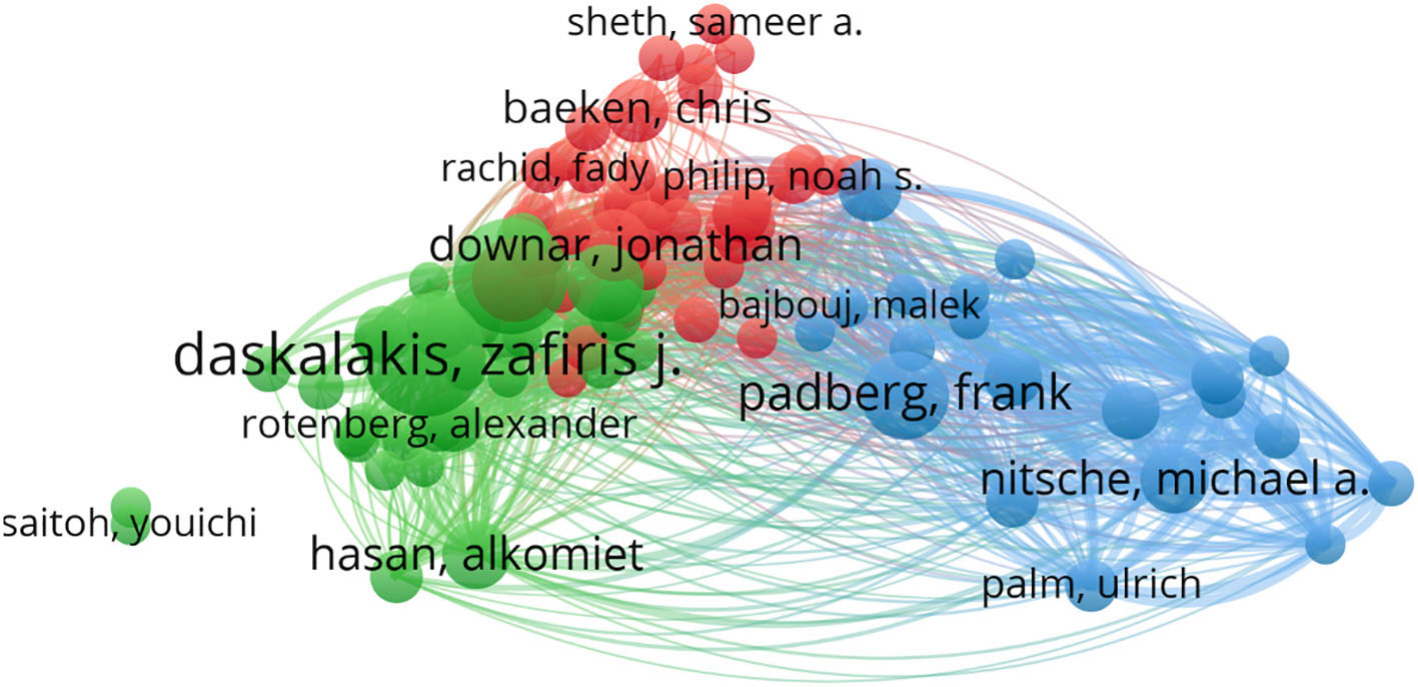
The cooperative network map of top 3 authors clusters on TMS for MD.

A total of 3,138 authors have contributed to research on TMS for MD. The **Table 2** shows the top 11 authors by publications and provides their relevant information. Daskalakis Zafiris J ranks first with 24 publications, followed by Fitzgerald Paul B with 21 and Padberg Frank with 13. These leading authors are primarily from Germany, Canada, and the USA. Among them, Nitsche Michael A from the Leibniz Research Centre holds the highest ranks in both total citations and average citations per paper. We use VOSviewer to generate a network visualization map, including authors with at least 2 publications. Out of the 315 authors who meet this criterion, 125 have no collaborations, while the remaining 190 have formed collaborative networks. These 190 authors are divided into 18 clusters, each represented by a distinct color. The clusters represented by red, green, and blue contain the most authors, each comprising 19 authors, centered on Daskalakis Zafiris, Downar Jonathan and Padberg Frank, as depicted in the **Figure 7**.

**Table 2:** The top 11 active authors who published literatures on TMS for MD.

Rank	Author	Country	Institution	Documents	Citations	Citations per paper
1	Daskalakis, zafiris. j	USA	University of California, San Diego	24	1294	53.92
2	Fitzgerald, paul. b	Australia	Monash University	21	1130	53.81
3	Padberg, frank	Germany	university of Munich	13	1494	114.92
4	Blumberger, daniel m.	Canada	University of Toronto	12	464	38.67
5	pascual-leon, alvaro	USA	Harvard Medical School	10	1255	125.50
6	Downer, jonathan	Canada	University of Toronto	9	962	106.89
7	Chen, robert	Canada	University of Toronto	8	472	59.00
8	Hasan, alkomiet	Germany	university of Munich	8	285	35.63
9	Nitsche, michael a	Germany	Leibniz Research Centre	8	1959	244.88
10	Baeken, chris	Belgium	Ghent University	7	141	20.14
11	Brunoni, andre r	Brazil	University de São Paulo	7	372	53.14

### 3.5 Journal analysis

**Figure 8:**
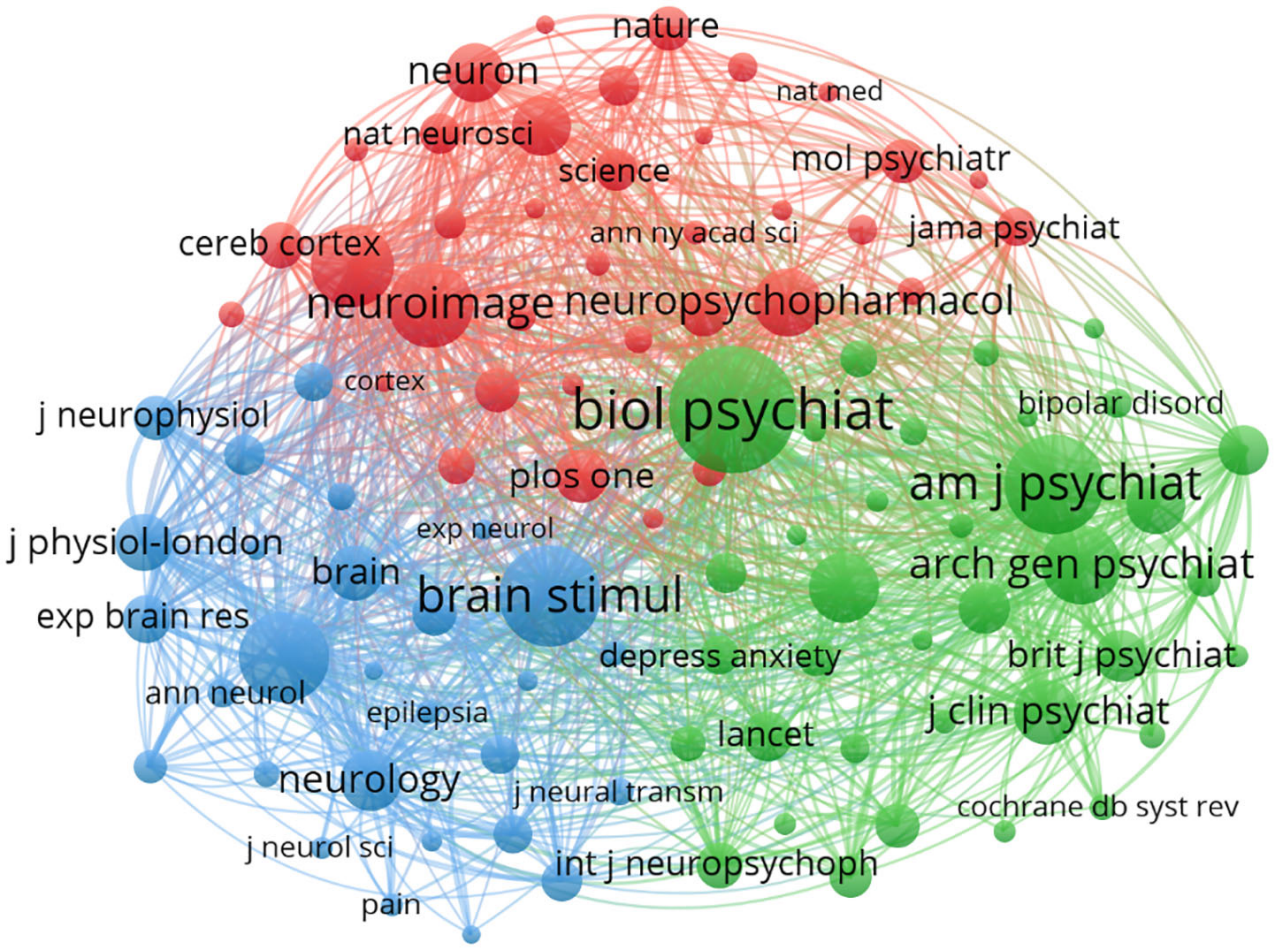
Co-citation network map of journals.

A total of 257 journals have published research in this field. The top 12 journals alone have published 152 papers, accounting for 24.88% of all research outputs, as listed in **Table 3**. The *Brain Stimulation* ranks first with 29 publications, followed by *Frontiers in Psychiatry* and *Frontiers in Human Neuroscience*, with 26 and 13 papers, respectively. Among these top 12 journals, 3 have an impact factor exceeding 5, while the others have impact factors ranging from 1.8 to 3.7. Additionally, 5 journals are classified in the Q1 category of the JCR rankings, another 5 in Q2, and 2 in Q3. To analyze co-citation patterns among these journals, we used VOSviewer to create a Co-citation visualization map, focusing on journals cited at least 100 times. As depicted in the **Figure 8**, the top 3 journals by co-citations are *Biological Psychiatry* (9.6), *American Journal of Psychiatry* (15.1), and *Brain Stimulation* (7.6), all of which are considered leading journals in this field.

**Table 3:** The top 12 most productive journals on TMS for MD.

Rank	Journal	Documents	Citations	Citations per paper	IF	JCR
1	Brain stimulation	29	1843	63.55	7.6	1
2	Frontiers in psychiatry	26	502	19.31	3.2	2
3	Frontiers in human neuroscience	13	254	19.54	2.4	2
5	Progress in neuro-psychopharmacology & biological psychiatry	11	446	40.55	5.3	1
6	Neuropsychiatric disease and treatment	10	333	33.30	2.5	2
7	Clinical neurophysiology	9	7961	884.56	3.7	1
8	Journal of ect	9	469	52.11	1.8	3
9	Journal of psychiatric research	9	472	52.44	3.7	2
10	European archives of psychiatry and clinical neuroscience	8	256	32.00	3.5	3
11	Frontiers in neuroscience	8	84	10.50	3.2	2
12	Neuropsychopharmaco- logy	8	645	80.63	6.6	1

### 3.6 Keywords analysis

**Figure 9:**
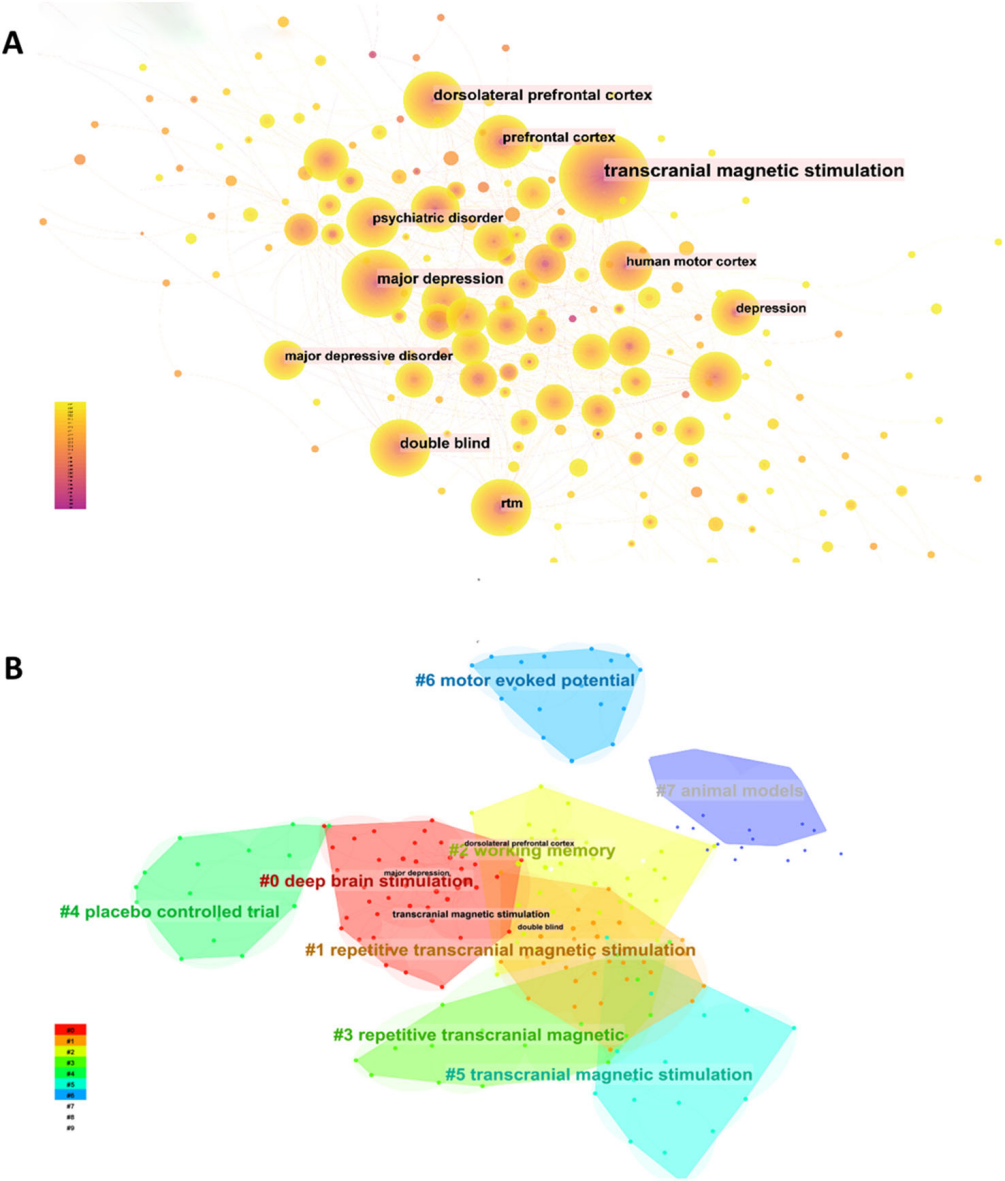
Analysis of keywords related to publications on TMS for MD. **(A)** The keyword co-occurrence network map. **(B)** The keyword cluster map.

Keywords serve as the core elements of an article, offering insights into current research hotspots and potential future directions of a discipline through co-occurrence analysis. As illustrated in the **Figure 9A** generated by citeSpace, the top 3 co-occurring keywords are “transcranial magnetic stimulation,” “major depression,” and “dorsolateral prefrontal cortex.” These keywords can be divided into 8 distinct categories, as showing in

**Figure 9B**: #0, #1, #3, and #5 correspond to the stimulation modes used in TMS applications for patients with MD. #2 and #6 function as reference indicators for TMS applications in MD, while #4 and #7 pertain to the types of experiments conducted in TMS research on MD.

**Figure 10:**
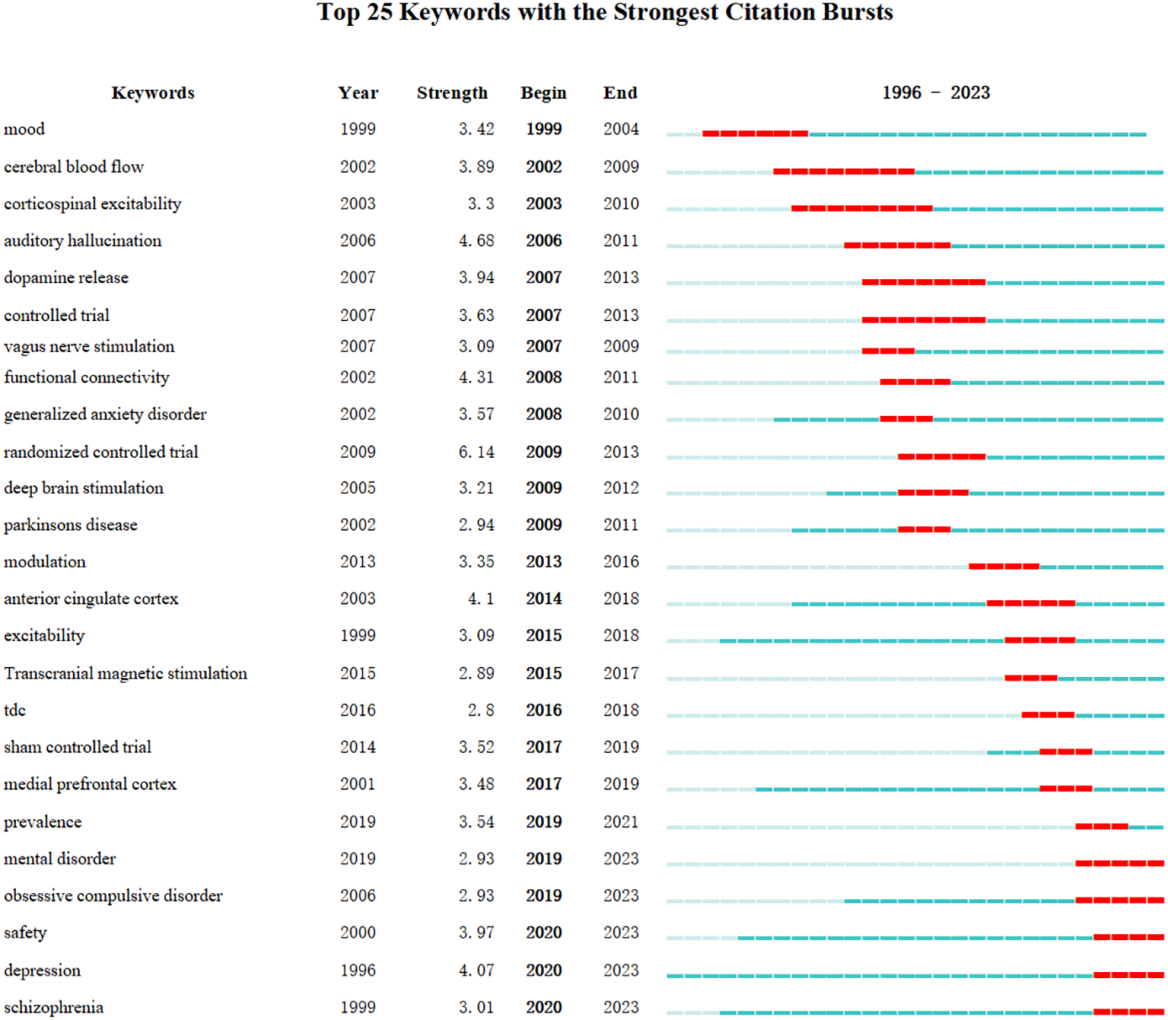
The top 25 keywords with the strongest citation bursts.

We utilized CiteSpace to generate the top 25 keywords with strongest bursts, as depicted in the **Figure 10**. The earliest burst terms, “Mood,” underscore the initial scholarly focus on emotional changes in mental disorder patients. “randomized controlled trail” exhibited the highest burst intensity, marking a critical turning point in the research trajectory of the field. The burst keywords “cerebral blood flow” and “corticospinal excitability” displayed the longest burst spans, highlighting the sustained attention these topics have garnered over time. More recently, “ depression,” “ obsessive compulsive disorder,” “ schizophrenia,” “ Mental disorder,” and “ safety “ have emerged as significant, continuing to attract attention as current research hotspots.

## 4 Discussion

### 4.1 Global trend on TMS for MD

Over the past few decades, MD and TMS have garnered significant attention from scholars worldwide, leading to an increasing number of related studies. This study reviews 611 articles on TMS treatment for MD and employs VosViewer, Citespace, and Scimago Graphica for visualization analysis to identify research hotspots and trends in the field. The **Figure 2** illustrates a steady growth trend in TMS treatment research from 1996 to 2023. Prior to 2008, the publication volume remained relatively stable, possibly because TMS technology was relatively underdeveloped and lack of recognition by experts in the field of MD. From 2008 to 2020, there was a notable upward trend in publications, peaking first in 2009 following the FDA’s approval of TMS for treating drug-resistant depression (23). A second peak in 2014 coincided with the release of the “Guidelines on TMS Treatment Based on Evidence-Based Medicine” by the International Federation of Clinical Neurophysiology (IFCN),which expanded the potential applications of TMS to a variety of MD (24). The third peak occurred in 2019, in line with the IFCN’s publication of updated TMS treatment guidelines (25), further highlighting TMS’s growing prominence in treating MD and expanding the recommended protocols for various indications. Since 2021, the publications have rapidly increased, surpassing 50 articles per year, indicating a heightened global interest in TMS as a safe, non-invasive neuromodulation for MD.

In the top 10 countries by publications, 8 are developed nations, while only China and Brazil are developing nations. Among these, the USA, Germany, and Canada are the primary contributors to this field. Representing developing nations, China entered the field later than the other top 10 countries. Despite this, it has demonstrated rapid progress over the past decade, becoming the third-largest research contributor after the USA and Germany. However, China’s low centrality score of 0.09 (**Figure 4A**), underscores its limited scientific collaboration with other nations in this field. Additionally, the network map of international institutional collaborations reveals that the top 10 research institutions in this field are predominantly from developed countries, including the University of Toronto in Canada, Harvard Medical School in the USA, Monash University in Australia, and King’s College London in the UK, with only Brazil’s University of Sao Paulo representing developing countries. The well-established university infrastructures and extensive academic resources of developed countries have led to a disparity in academic exchanges between developed and developing countries. This imbalance can be attributed to several factors. Developed countries began investigating the potential of this field earlier than developing countries, with scholars from Australia and Germany exploring the possibility of using TMS to treat depression as early as the 1990s (2, 26). In contrast, developing countries like China have only recently begun to establish collaborative networks and publish significant research findings in the last decade. Moreover, the lack of sufficient funding and attention toward TMS treatment for MD in developing countries may hinder their ability to produce high-quality research outcomes.

As indicated in **Table 2**, nearly all of the top 11 authors are affiliated with research institutions in developed countries. Professor Zafiris Daskalakis, from the University of California, San Diego, USA, is ranked as the top 1 author. As an internationally recognized expert, Professor Daskalakis specializes in using TMS to treat severe MD. His research team focuses extensively on the application of TMS in treating conditions such as treatment-resistant depression (TRD), schizophrenia, suicidal thoughts, obsessive-compulsive disorder, and explores its use in cognitive neuroscience. They also delve into the neurophysiological principles of using TMS and magnetic seizure therapy (MST) for depression treatment (27–33). The author collaboration clustering map reveals three primary clusters centered around key researchers: Zafiris Daskalakis with Paul Fitzgerald, Frank Padberg with Michael Nitsche, and Jonathan Downar with Chris Baeken. Daskalakis and Fitzgerald concentrate on the neurophysiological basis of TMS for depression and posit that TBS could serve as a superior intervention for TRD (34). Padberg and Nitsche investigate how smoking affects cortical excitability in schizophrenia patients, aiming to understand the high prevalence of smoking in this group (35). Lastly, Downar and Baeken explore the potential of accelerated repetitive rTMS to enhance therapeutic outcomes and shorten the duration of treatment for severe depression (36).

The publication ranking (**Table 3**) and co-citation analysis (**Figure 8**) reveal that Brain Stimulation is the most influential journal in this field and a leading journal in neuromodulation, with an impressive impact factor of 7.6 in 2024. Additionally, the top 12 journals together account for less than one-third of total publications, demonstrating that research on TMS treatment for MD is extensively distributed across a variety of journals. Notably, *Brain Stimulation* is the sole journal featured in both the top 12 for publications and the top 3 for citation analysis, where the impact factors of journals in the top 3 exceed those in the top 10 for publications. This disparity suggests that the overall quality and standard of research on TMS treatment for MD might be relatively low, emphasizing the need for more rigorous international collaboration and enhanced quality of research in this field.

### 4.2 Hotspots and emerging frontiers analysis

In bibliometrics, commonly used keywords highlight major themes and emerging patterns, crucial for understanding the evolution of the field. These keywords offer valuable insights into future research directions and emerging trends. A significant citation burst in a keyword, for instance, may indicate a potential future trajectory for TM applications in MD research. This summary utilizes keyword analysis to provide an overview of the current hotspots and trends in TMS applications for MD.

#### 4.2.1 TMS for depression

##### 4.2.1.1 rTMS for depression

In 1995, George (37) et al. published the first study on TMS for treating depression in Neuroreport, which noted that daily rTMS stimulation of the left frontal cortex could alleviate symptoms in patients with medication-resistant depression (MRD). Subsequent research confirmed the therapeutic effects and safety of TMS for treatment-resistant depression (TRD), with a noticeable increase in related studies. Between 2001 and 2011, over ten peer-reviewed meta-analyses and qualitative reviews supported the efficacy of high-frequency rTMS targeting the left dorsolateral prefrontal cortex (L-DLFPFC) (38–40),which was grounded in imaging studies that linked this specific brain area with emotional dysregulation (41). Functional neuroimaging studies have shown the prefrontal cortex’s capacity to regulate emotions and affective behaviors, with high-frequency rTMS targeting the L-DLPFC yielding the most effective antidepressant outcomes (42). However, Yu (43) et al. discovered that increasing the dosage (total pulse count) and duration of rTMS treatments did not consistently improve symptom mitigation in TRD patients, as the dose-response relationship was influenced by both the stimulation frequency and the patient’s age. Moreover, the antidepressant efficacy of high-frequency rTMS targeting the L-DLFPFC was significantly enhanced over sessions ranging from 5 to 20, with a total of 1200 to 1500 pulses per day proving most effective (44). Nonetheless, the use of high-frequency rTMS on the L-DLFPFC also raised concerns about potential adverse events such as mania, seizures, and fainting (45). Consequently, for patients unresponsive to medication and intolerant to high-frequency-rTMS of the L-DLFPFC, low-frequency rTMS targeting the right DLPFC (R-DLPFC) might be a better alternative (46).

##### 4.2.1.2 deep-TMS (dTMS) for depression

As TMS technology and equipment have advanced, dTMS has emerged as a novel (47), non-invasive neuromodulation technique increasingly used in the treatment of severe depression (48). In 2009, Levkovitz et al. (49) conducted the first study using an “H” shaped coil on patients with medication-resistant major depressive disorder (MDD), applying 20Hz high-frequency stimulation at 120% RMT (Rest Motor Threshold) intensity for unilateral stimulation, which significantly improved depressive symptoms. In 2011, Isserles et al. (50) assessed the safety and effectiveness of “H1” shaped dTMS as an adjunct therapy for treatment-resistant unipolar depression, revealing its effectiveness for patients both with and without pharmacological treatments. Bersani et al. (51) ‘ comparative review observed that dTMS outperforms standard TMS in treatment efficacy and exhibits good tolerability. Moreover, dTMS significantly enhances cognitive functions, while ECT (Electroconvulsive Therapy), though effective, necessitates general anesthesia and could lead to serious side effects.

Research also shows that dTMS significantly alleviates the severity of depression and anxiety, with response rates ranging from 38% to 55% (52, 53). It can stimulate to a depth of 6 cm (54), impacting several brain structures related to depression, including the dorsolateral and dorsomedial prefrontal cortex, amygdala, hippocampus, ventral striatum, anterior and subgenual cingulate cortex, and posterior orbitofrontal cortex (55). It is hypothesized that through deeper stimulation, dTMS may achieve more effective therapeutic outcomes. Additionally, combining antidepressant drugs with dTMS has been found to enhance efficacy, suggesting a potential synergistic effect at the neurobiological level (56–58).

##### 4.2.1.3 TBS for depression

TBS is a highly efficient, patterned form of rTMS designed to modulate cortical activity (59). TBS rapidly influences synaptic plasticity, offering advantages over traditional rTMS, including shorter stimulation durations, enhanced cost-effectiveness, and an improved patient experience (29, 60). In 2018, Blumberger et al. (29) demonstrated that iTBS was non-inferior to 10 Hz rTMS in treating TRD over a 4–6 week period, although it was associated with slightly higher reported pain (mean score of 3.8 vs. 3.4 on a 10-point scale). Their research team further confirmed in a subsequent trial that a 4-minute bilateral TBS protocol was equivalent in efficacy to a 47.5-minute bilateral rTMS intervention for older adults with TRD over the same 4–6 week timeframe (59). Bulteau et al. (60) reported that iTBS yielded superior response and remission rates during a 4–6 week intervention, with effects sustained for up to 6 months, underscoring its time- and cost-efficiency. However, Fitzgerald et al. (61) found no significant differences in outcomes between iTBS and rTMS at 16 weeks, noting that depressive symptoms continued to decrease over 26 weeks for both approaches. This suggests that while iTBS reduces individual session duration, the total number of sessions remains comparable to rTMS, and variations in efficacy may depend more on patient-specific responses than on stimulation protocols alone. In clinical practice, a 4–6 week treatment course may warrant consideration.

##### 4.2.1.4 aTMS for depression

Accelerated TMS (aTMS) offers an innovative approach to managing TRD by condensing treatment into fewer days, which is particularly advantageous for patients requiring remote care or those with demanding schedules, thereby improving treatment compliance (62, 63).

In contrast to rTMS, which typically involves 20–30 sessions delivering 1200–3000 pulses over 4–6 weeks, or intermittent theta-burst stimulation (iTBS), which entails 20 sessions over a similar duration, aTMS protocols significantly compress the treatment timeline. Examples include 45 iTBS sessions administered over 15 weekdays or 50 sessions (10 daily) delivered across 5 days, providing thousands of pulses rapidly for TRD or bipolar depression (BD) (64, 65). Fitzgerald et al. (66) found no significant difference in efficacy between a 3-week aTMS protocol and a 4-week standard rTMS regimen for major depressive disorder (MDD), although aTMS was associated with increased discomfort. Similarly, Ramos et al. (65) reported higher response and remission rates with 45 aTMS sessions compared to sham treatment, albeit with more frequent scalp pain. Appelbaum et al. (64) and Sheline et al. (67) observed superior symptom reduction in BD with a 5-day accelerated iTBS (aiTBS) protocol. Compared to the single daily sessions of iTBS, the multi-session-per-day structure of aTMS intensifies treatment delivery but heightens discomfort. This suggests that while a reduced treatment duration enhances accessibility, the increased session frequency and pulse intensity may compromise tolerability. However, comparative studies between aTMS and other TMS modalities for MDD remain limited, highlighting the need for further optimization of session frequency and intervals relative to rTMS and iTBS.

#### 4.2.2 TMS for obsessive-compulsive disorder

Obsessive-Compulsive Disorder (OCD) mainly manifests as irrepressible repetitive impulses and specific actions (68), commonly attributed to abnormalities in the cortico-striato-thalamo-cortical (CSTC) circuit (69), which is involved in emotional, cognitive, and motor control. These abnormalities can result in deficits in information processing and response control. Modifying the activity in these neural circuits is anticipated to develop into an effective treatment method (70).

##### 4.2.2.1 Targeting DLPFC

The DLPFC is closely associated with cognitive control (71), and its dysfunction may lead to emotional and behavioral problems such as excessive worry, doubt, guilt, and repetitive behaviors, which are typical symptoms of OCD (72). Given its crucial role, the DLPFC is a primary target for TMS to OCD patients (73). Research indicates that TMS treatment targeting the DLPFC can significantly lower the symptom scores on the Yale-Brown Obsessive Compulsive Scale (Y-BOCS) in OCD patients, particularly when applying bilateral or right-side low-frequency stimulation (70). Nevertheless, the variability in stimulation frequency and technique across studies suggests the need for further research to determine the most effective treatment protocol (74).

##### 4.2.2.2 Targeting mPFC and ACC

Recently, the medial prefrontal cortex (mPFC) and anterior cingulate cortex (ACC) have been identified as new targets for dTMS in treating OCD, using specialized devices such as double cone coils and “H7” coils, and showing positive effects in these brain regions (75, 76). The success of dTMS might stem from its ability to simultaneously stimulate the mPFC and ACC, leading to neurobiological improvements in areas including thinking, motivation, emotional and action integration, pre-movement response selection, error monitoring, and cognitive conflict detection (77–79). This indicates that dTMS could surpass the limitations of traditional rTMS by affecting deeper subcortical neural pathways. Therefore, dTMS could become a promising alternative treatment for OCD patients who have not responded well to conventional medication and psychological therapies (80), but the body of research on TMS remains limited, and further studies are essential to confirm these findings.

##### 4.2.2.3 Targeting SMA

Neurophysiological studies suggest that motor “intrusions” and repetitive behaviors in OCD patients are likely caused by reduced cortico-subcortical inhibition and increased cortical excitability (81). Paired pulse TMS research has shown diminished intracortical inhibition in OCD patients, particularly noting lower resting and active motor thresholds in the left hemisphere (82). Mantovani (83) et al. conducted low-frequency rTMS on the bilateral supplementary motor area (SMA) in OCD patients and observed reduced hyperexcitability in the right hemisphere and restored symmetry of motor thresholds, alongside significant improvements in OCD symptoms. This supports the SMA as an effective TMS target in treating OCD. Lee et al. (84) found that 1Hz low-frequency stimulation of the SMA specifically alleviates compulsive behaviors, likely due to the SMA’s regulatory role over subcortical regions and its impact on OCD symptoms. Rehn et al. (85) performed a meta-analysis of 18 randomized controlled trials, demonstrating that low-frequency TMS stimulation of the bilateral SMA regions yielded the most favorable outcomes. Additionally, Yu et al. (86) reviewed 26 studies in a rapid systematic analysis and concluded that low-frequency stimulation of the SMA is potentially more effective than that of the DLPFC. This efficacy might stem from the stronger connectivity of the SMA with the striatum compared to that of the DLPFC, which temporarily “normalizes” the interactions between the SMA and the CSTC circuit. This normalization aids patients in more effectively managing intrusive thoughts and controlling impulsive behaviors (87).

##### 4.2.2.4 Targeting OFC

Patients with OCD effectively manage their symptoms using selective serotonin reuptake inhibitors (SSRIs), cognitive-behavioral therapy (CBT), and deep brain stimulation (DBS) (88, 89). PET scans reveal that these treatments significantly reduce the elevated metabolic activity in the orbitofrontal cortex (OFC) within the CSTC circuit. This finding affirms the OFC’s role as a crucial neural area in OCD and as a marker of treatment response (90). As a result, the OFC has emerged as a significant target for TMS. However, Liu et al. (91) demonstrated that cTBS using a **Figure 8** coil on the right OFC did not alleviate symptoms in treatment-resistant OCD patients. In contrast, Dutta (89) et al. reported that cTBS on the left OFC in patients with moderate to severe OCD led to improvements in anxiety symptoms and overall severity. Given the OFC’s deep location, using double cone coils or “H7” coils may be more effective for dTMS treatments. Additionally, Nauczyciel (90) et al. suggest that low-frequency dTMS stimulation of the OFC primarily enhances the management of acute OCD symptoms, serving as a supplementary method alongside SSRIs and CBT (92).

#### 4.2.3 TMS for schizophrenia (SCZ)

Schizophrenia (SCZ) is a complex mental disorder characterized by severe impairments in cognitive, clinical, and psychosocial functions (93, 94). It includes positive symptoms such as auditory verbal hallucinations, disorganized speech, and delusions, as well as negative symptoms like affective flattening, attention impairment, alogia, avolition-apathy, and anhedonia-asociality (95–97).While only approximately 50% of patients respond to antipsychotic medications with some improvement in positive symptoms, these drugs have limited efficacy for negative symptoms and cognitive impairments (98). Due to these limitations, alternative therapeutic approaches are being explored. Since Klein (99) et al. first reported the use of TMS in treating schizophrenia in 1999, there has been a growing interest among researchers in this field.

##### 4.2.3.1 TMS for positive symptoms of SCZ

Neurophysiological studies indicate that during persistent auditory hallucinations in patients with SCZ, there is abnormal hyperactivity in the speech processing areas of the bilateral temporal lobes (100). To counteract this overactivation, Slotema et al. (101) conducted a comprehensive review of 25 randomized controlled trials and found that 1 Hz low-frequency rTMS directed at the left temporoparietal cortex can effectively reduce this hyperactivity. This treatment modality is currently considered potentially the most effective TMS approach for managing auditory hallucinations and other positive symptoms.

##### 4.2.3.2 TMS for cognitive and negative symptoms of SCZ

In patients with SCZ, cognitive processes—particularly working memory—are supported by gamma oscillations at approximately 40 Hz (102). However, electrophysiological and anatomical research has revealed that these patients have disrupted neuronal oscillation synchrony and abnormal neurotransmitter transmission, leading to an excitation-inhibition imbalance, adversely affecting brain functional networks and contributing to both cognitive and negative symptoms (103). Several meta-analyses have indicated that high-frequency (10 Hz) rTMS targeting the L-DLPFC, at intensities exceeding 100% of the AMT (Active Motor Threshold), and administered for at least three weeks, might be the most effective protocol for alleviating negative symptoms, although its efficacy remains moderate (effect sizes 0.49 to 0.64) (104–106). Recent studies have demonstrated that iTBS targeting prefrontal connections within the cerebellum can enhance the treatment of negative symptoms, suggesting a promising new direction for further research into cerebellar iTBS’s role in treating these symptoms (107).

In general, the treatment of schizophrenia requires different strategies for various symptoms and functional impairments, with TMS and dTMS presenting new therapeutic options.

#### 4.2.4 TMS for other MD

Clinical evidence, expert guidelines, and regulatory approvals support the use of TMS in treating various MD. Specifically, for substance abuse and addiction issues such as smoking, alcoholism, and drug addiction, high-frequency rTMS targeting the L-DLPFC or bilateral DLPFC and insula stimulation using high-frequency dTMS is considered to have anti-addictive effects (108). For instance, a combination of cognitive-behavioral therapy with high-frequency dTMS targeting the unilateral DLPFC and bilateral insula has achieved a 44% abstinence success rate among patients with nicotine addiction (109). Additionally, patients addicted to methamphetamine have shown reduced craving symptoms and improved cognitive functions after receiving high-frequency rTMS on the L-DLPFC (110, 111). However, there is currently no consensus on specific TMS protocols for effectively treating substance addiction due to insufficient evidence.

TMS also shows potential for treating generalized anxiety disorder (GAD). Studies demonstrate that 1Hz low-frequency rTMS of the right parietal lobe can effectively alleviate symptoms of anxiety and insomnia in GAD patients (112). Additionally, applying 1mHz infra-low frequency TMS (ILF-TMS) across the entire brain could serve as a promising adjunctive treatment for reducing anxiety symptoms in GAD patients (113). Croarkin (114) et al. in a series of case studies, discovered that 1Hz low-frequency aTMS of the dorsomedial prefrontal cortex (DMPFC) also effectively improves symptoms in young and middle-aged patients with anxiety disorders. However, Parikh (115) et al. reviewed six studies for a meta-analysis, and noted that given the limited and heterogeneous nature of the research, rTMS has a notable impact on GAD. This finding underscores the urgent need for well-designed, randomized controlled trials to further explore the treatment of GAD and related anxiety disorders.

TMS has become increasingly recognized for its efficacy in treating Post-Traumatic Stress Disorder (PTSD), where high-frequency TMS stimulation of the R-DLPFC has proven more effective (24), with benefits persisting up to three months (116). Simultaneously, low-frequency TMS applied to the right prefrontal cortex, combined with Cognitive Processing Therapy (CPT), has effectively reduced panic symptoms in PTSD patients, maintaining these effects for six months (117). Additionally, high-frequency stimulation of the L-DLPFC is linked to significant emotional improvements in these patients (116). A recent study has shown that both iTBS and high-frequency rTMS stimulation of the R-DLPFC yield similar results in reducing anxiety symptoms in PTSD patients, though iTBS offers the advantage of a shorter treatment period (118). TMS has also been applied in treating other mental health conditions, such as post-stroke depression (119), attention deficit hyperactivity disorder (ADHD) (120), autism spectrum disorder (ASD) (121), and mental developmental delays (122). Nevertheless, due to the lack of adequate or consistent evidence, regulatory authorities have yet to approve these treatments, and there are no formal recommendations in expert guidelines.

#### 4.2.5 Safety analysis of TMS application in MD

The safety of TMS, as its use expands in treating various MD, is increasingly scrutinized by the global academic community. DC. Nahas (123) first investigated the safety of TMS in pregnant patients with depression in 1999. Over subsequent decades, new stimulation protocols have been developed, consistently prioritizing safety in clinical settings. There has been a trend in updating stimulation protocols for MDD patients that involves increasing stimulation intensity to enhance treatment efficacy. For instance, typical TBS intensities range from 80%-120% of the RMT, with increments to 80% AMT, 80% RMT administered twice daily, 120% RMT five times daily, and exceeding 120% RMT (124–127). The most recent aTMS mode employs the highest stimulation intensity. Kevin (128) et al. reviewed 85 studies and observed that the seizure incidence with aTMS (0.0023%) is comparable to that of standard TMS (0.0075%). Other side effects reported include acute headaches, fatigue, and scalp discomfort, demonstrating that aTMS maintains robust safety and patient tolerance under various conditions.

Despite the ongoing development and application of TMS, the lack of detailed research into its dose-response relationship means it remains uncertain whether higher intensities or more frequent stimulations provide greater efficacy than standard TMS protocols. Theoretically, using protocols with lower intensities could be safer, an idea that merits further investigation. Reports indicate that patients with depression treated with high-frequency rTMS may experience hypomanic symptoms, including insomnia, restlessness, or anxiety (129). A comprehensive systematic review and meta-analysis (130) encompassing 53 studies with 3273 patients identified non-serious adverse events associated with the treatment, such as transient headaches, discomfort, pain, and tinnitus. These side effects are quickly resolvable or can be effectively managed with medication after ceasing TMS or adjusting its parameters.

A critical concern in TMS therapy is the risk of seizure induction, which, while exceedingly rare, constitutes the most severe adverse effect associated with this treatment. Research suggests that patients with MD such as depression and SCZ exhibit an inherently elevated baseline risk of seizures (131–134), a vulnerability that may be exacerbated by stressors such as sleep deprivation and psychological stress (135–137). Notably, Tendler et al. (138) reported that, among 31 documented seizure cases, 26 occurred in patients with depression treated with dTMS using an “H1” coil, indicating that this specific dTMS modality may carry a heightened propensity for seizure induction. Furthermore, high-frequency TMS protocols, which typically involve stimulation at frequencies exceeding 10 Hz, have been associated with an increased risk of adverse effects, especially about seizures (139). Studies suggest that the rapid delivery of pulses in high-frequency regimens may overstimulate cortical networks, potentially lowering the seizure threshold, particularly in susceptible individuals (140). However, comparative analyses evaluating seizure risks across different TMS protocols, including high-frequency variants, remain limited. TMS is frequently employed as an adjunct to pharmacotherapy for MD, with studies by Cao (141) and Blumberger (127) demonstrating that adjunctive TMS therapy does not significantly elevate the incidence of adverse effects. International guidelines affirm that standard TMS procedures are associated with a low overall incidence of adverse events. Nevertheless, given the potential for severe reactions, particularly with high-frequency protocols or specific modalities like dTMS, it is essential to thoroughly inform patients of these risks during clinical implementation.

## 5 Conclusion

This paper utilizes bibliometric techniques to delve deeply into the application of TMS for MD over the past three decades. Research trends indicate a consistent increase, with North America and Europe leading in terms of publication output and total citation counts. This analysis extends to international collaborations and current trends in the field, highlighting a notable point: many journals in this area do not have high impact factors, suggesting a need for increased academic focus moving forward. We have summarized TMS treatment protocols that are recommended by international clinical guidelines and experts for conditions such as treatment-resistant depression, obsessive-compulsive disorder, schizophrenia, and general anxiety disorders. Some promising TMS protocols, not yet recognized in these guidelines, necessitate validation through more extensive multicenter clinical trials to verify their efficacy. Additionally, we conduct a safety review of TMS in mental health applications, confirming that its usage remains within safe operational boundaries. The paper also outlines several pressing clinical challenges in applying TMS to MD, the resolution of which could significantly aid researchers in better understanding the evolving focal points and future directions of this field. This involves leveraging advancements in neurophysiology, neuroimaging, and neuroscience to find new indications and stimulation targets, optimize protocols, discover markers of treatment response, and explore the neurophysiological mechanisms of various TMS modes as symptoms progress.

## Data Availability

The original contributions presented in the study are included in the article/supplementary material. Further inquiries can be directed to the corresponding author.
